# Resistance to Fluoroquinolones in *Pseudomonas aeruginosa* from Human, Animal, Food and Environmental Origin: The Role of *CrpP* and Mobilizable ICEs

**DOI:** 10.3390/antibiotics11091271

**Published:** 2022-09-19

**Authors:** María López, Beatriz Rojo-Bezares, Gabriela Chichón, Yolanda Sáenz

**Affiliations:** Área de Microbiología Molecular, Centro de Investigación Biomédica de La Rioja (CIBIR), 26006 Logroño, Spain

**Keywords:** *Pseudomonas*, ciprofloxacin, mutational resistome, efflux pump, CrpP, one health

## Abstract

Fluoroquinolone resistance and the associated genetic mechanisms were assessed by antimicrobial susceptibility and whole genome sequencing in 56 *Pseudomonas aeruginosa* strains from human, animal, food and environmental origins. *P. aeruginosa* PAO1, PA7 and PA14 reference strains were also included in the study. Twenty-two strains (37%) were resistant to, at least, one fluoroquinolone agent. Correlation between the number of changes in GyrA and ParC proteins and the level of fluoroquinolone resistance was observed. Mutations or absence of genes, such as *mexZ*, *mvaT* and *nalD* encoding efflux pumps regulators, were also found in resistant strains. The *crpP* gene was detected in 43 strains (72.9%; 17 of them non-clinical strains), and coded seven different CrpP variants, including a novel one (CrpP-7). The *crpP* gene was located in 23 different chromosomal mobile integrative and conjugative elements (ICEs), inserted in two tRNAs integration sites. A great variety of structures was detected in the *crpP*-ICEs elements, e.g., the fimbriae related *cup* clusters, the mercury resistance *mer* operon, the pyocin S5 or S8 bacteriocin encoding genes, and mobilization genes. The location of *crpP*-like genes in mobilizable ICEs and linked to heavy metal resistance and virulence factors is of significant concern in *P. aeruginosa*. This work provides a genetic explanation of the fluoroquinolone resistance and *crpP*-associated pathogenesis of *P. aeruginosa* from a One-Health approach.

## 1. Introduction

*Pseudomonas aeruginosa* is an environmentally ubiquitous species, with great metabolic versatility and with an extraordinary ability to inhabit animals, soil, water or plants, from which it is easily transmissible. *P. aeruginosa* is a versatile opportunistic pathogen of great clinical importance due to its high pathogenicity and antimicrobial resistance [1].

Fluoroquinolones are synthetic products of broad-spectrum antimicrobial action, and are a major class of antibiotics used to treat *P. aeruginosa* infections. Their mechanism of action is the inhibition of DNA gyrase and topoisomerase IV, causing DNA breakdown and cell death [2]. However, bacterial resistance to fluoroquinolones has progressively been increased [2,3]. Three main resistance mechanisms have been detected in *P. aeruginosa*: 1) Chromosomal point mutations in the so-called quinolone resistance determining region (QRDR) of the genes encoding GyrA and GyrB subunits of the DNA gyrase, and ParC and ParE subunits of the topoisomerase IV. The amino acid positions more frequently described are Thr83 and Asp87 in GyrA, Ser466 and Glu468 in GyrB, Ser87 in ParC, and Asp419, Glu459, Ala473 and Ser457 in ParE [3]. 2) Efflux pumps overexpression or permeability alterations that reduce the intracytoplasmic concentration of fluoroquinolones. Four efflux pumps of *P. aeruginosa* are known to efflux fluoroquinolones (MexCD-OprJ / MexEF-OprN / MexAB-OprM / MexXY-OprM), and their overexpression is associated with mutations in their regulator genes (*nfxB / mexS / mexR, nalC, nalD / mexZ*, respectively) [3]. 3) Acquisition of transferable mechanisms of quinolone resistance (TMQR), such as Qnr, QepA, OqxAB, AAC(6′)-Ib-cr, QacA and QacB families [2]. TMQR prevalence is low in *P. aeruginosa* [3,4,5].

In addition, a 65-amino-acid protein, CrpP (ciprofloxacin resistance protein, plasmid encoded), was first reported in 2018 as a novel ciprofloxacin-modifying enzyme, encoded by a gene located on the pUM505 plasmid in a clinical *P. aeruginosa* isolate [6]. Thereafter, abundant *crpP* homologues have been reported in *P. aeruginosa* and in *Enterobacteriaceae* [4,7,8,9,10]. *crpP* genes have been identified in *P. aeruginosa* as part of different mobile integrative and conjugative elements (ICEs) that frequently transfer horizontally. Moreover, genetic environment of *crpP* gene could carry different resistance genes, virulence factors and various mobile genetic elements that may contribute to the spread of pathogens in hospital settings [4,8,11].

The majority of fluoroquinolone-resistance and CrpP studies have focused on clinical *P. aeruginosa* strains from humans, but scarce or absent on animal, food and environmental strains. This study aimed to characterize the fluoroquinolone resistance in a collection of *P. aeruginosa* from different origins, and to assess the associated genetic resistance mechanisms.

## 2. Results and Discussion

**Figure 1:**
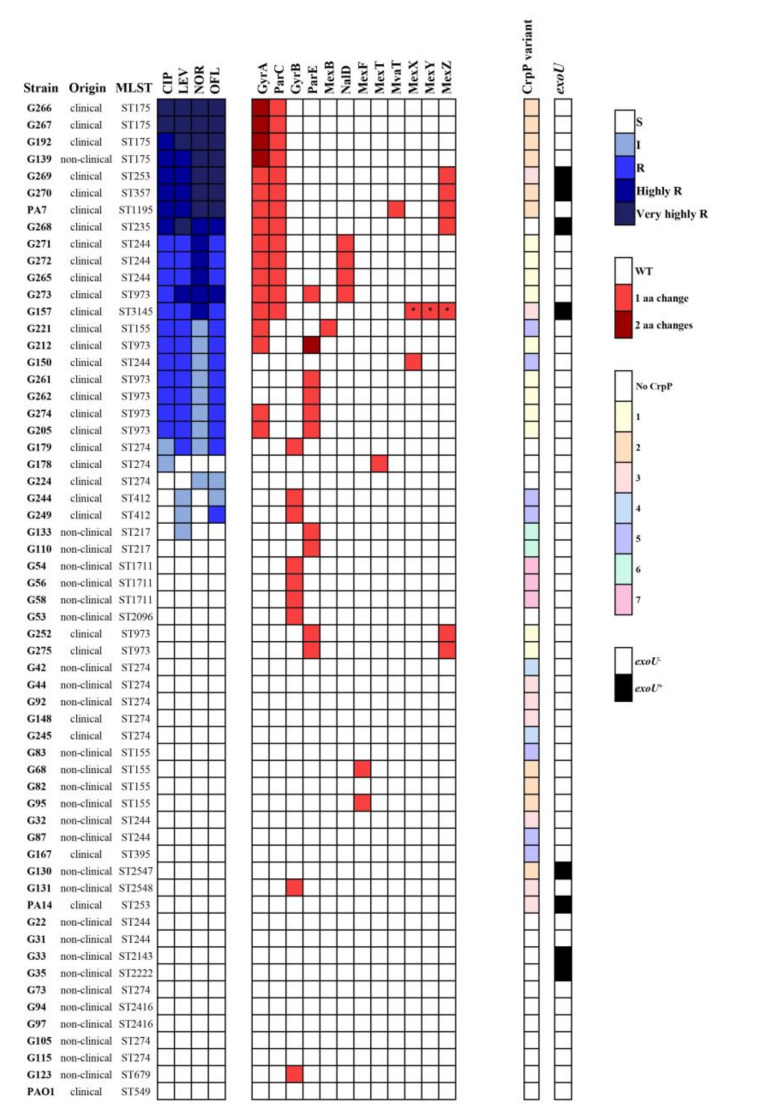
Heatmap Showing Minimum Inhibitory Concentrations (MIC) of Fluoroquinolones (CIP = ciprofloxacin; LEV = levofloxacin; OFL = ofloxacin; NOR = norfloxacin) Where S Corresponds to Susceptible, I to Intermediate, R to Resistant until 32mg/L, highly R from >32 to 128mg/L, and very Highly R >128mg/L (levels of resistance according to Clinical and Laboratory Standards Institute, CLSI [13]). The different variants of CrpP are shown in different colors, and the presence of the *exoU* gene is indicated in black. The fluoroquinolone relevant alterations detected in 12 chromosomal proteins are shown in red or maroon colors depending on the number of alterations. *P. aeruginosa* PA14 is the same as UCBPP-PA14. * Protein not detected.

The high use of fluoroquinolone agents has contributed to the selection and dispersion of fluoroquinolone-resistant pathogens in both hospital and environmental settings. In this study, the resistance to fluoroquinolones and the involved genetic mechanisms have been analyzed in a collection of 56 *P. aeruginosa* strains isolated from a wide variety of clinical and non-clinical origins, including clinical and healthy humans, healthy animals, water, and food (Table S1). In addition, the three *P. aeruginosa* PAO1, PA14, and PA7 reference strains were also included in the study. Thirty-three strains (55.9%), including *P. aeruginosa* PAO1 and PA14, were susceptible to all fluoroquinolones tested (ciprofloxacin, levofloxacin, norfloxacin and ofloxacin). All but two non-clinical strains showed this susceptible phenotype (Figure 1, and Table S2). On the other hand, 22 strains (37.3%) were resistant to at least one fluoroquinolone tested, being twelve of them and *P. aeruginosa* PA7 resistant to all fluoroquinolones tested (22%). These resistance percentages were slightly higher than those detected in invasive (blood or cerebrospinal fluid) isolates in Spain (18.1%) and Europe (18.9%) [12]. 

The three main fluoroquinolone resistance mechanisms have been studied in the 59 *P. aeruginosa* strains of our study: chromosomal mutations in drug targets; efflux pump activity or permeability alterations; and acquisition of TMQR. The mutational resistome was determined by analyzing the mutations in 23 chromosomal genes implicated in fluoroquinolone targets and efflux pumps (Table S3).

### 2.1. Chromosomal Mutations in Fluoroquinolone Targets

Regarding the amino acid changes detected within the QRDR of GyrA, ParC, GyrB and ParE: two different substitutions were identified in GyrA (S83I and D87N), and ParE proteins (H461D and A473V), and three changes were observed in GyrB (S466A, S466F and I529V) and ParC (S87W, S87L and L95Q) (Table S2). To our knowledge, this is the first report of the L95Q substitution in the ParC protein of *P. aeruginosa* strains, and it was detected in a fluoroquinolone-resistant clinical strain (*P. aeruginosa* G273). No single ParC changes were found in our *P. aeruginosa* collection, whereas single GyrB and ParE substitutions were detected among ciprofloxacin-susceptible and resistant strains. As in previous studies [14,15,16], a correlation between the number of changes in GyrA and ParC proteins and the level of fluoroquinolone resistance was observed. None fluoroquinolone-susceptible *P. aeruginosa* showed changes either in QRDR of GyrA or ParC. A single amino acid change in the GyrA protein was associated with low ciprofloxacin resistance (MIC 2–4 mg/L), two substitutions (one in GyrA and one in ParC) with a moderate or high level of ciprofloxacin resistance (MIC 8–128 mg/L), and three substitutions (two in GyrA and one in ParC) were associated with the highest ciprofloxacin MICs (128–256 mg/L) (Figure 1, and Table S2). Curiously, all strains belonging to ST175 showed the highest fluoroquinolone MICs and the same substitutions in GyrA (T83I+D87N), and in ParC (S87W+L168Q).

Recently, Rehman et al. (2021) [15] highlighted the importance of gene–gene interactions in ciprofloxacin resistance. They showed that the T83I substitution in GyrA (without an additional mechanism of resistance) is unable to confer a ciprofloxacin-resistance in *P. aeruginosa*, being 1 mg/mL the predicted ciprofloxacin MIC. They also observed that the presence of other mutations (in GyrB, ParE, ParC or NfxB) increases the MIC only if the GyrA mutation is present. This is in accordance with our results (Figure 1, and Table S2), whereas there were strains that did not follow the above correlation pattern, such as the three ciprofloxacin-resistant strains (G150, G261, G262) without mutations in GyrA and ParC; or the 9 strains that show a wide range of ciprofloxacin MIC (MIC 8-128 mg/mL) although they harbored the same amino acid substitutions (T83I in GyrA and S83L in ParC) (Figure 1, and Table S2). The presence of active efflux pumps or mutations that affect regulatory genes could explain these situations as it is showed below.

### 2.2. Efflux Pumps Overexpression and Mutational Resistome

PAβN is a widely used efflux-pump inhibitor in *Enterobacteriaceae* and *P. aeruginosa*, where a four- or eight-fold reduction in the fluoroquinolone MIC in the PAβN presence is an indicator of an efflux-pump overexpression [17,18]. In our study, 38 strains reduced their ciprofloxacin MIC by more than two-fold in presence of 40 mg/L PAβN (Table S2), 17 of them being ciprofloxacin-resistant strains (MIC reduction range from 4 to > 233 folds). This effect was especially noticeable in strains with one single GyrA substitution and in the G157 strain, because the MIC of ciprofloxacin decreased from resistant to susceptible levels. Besides, the nine strains (including G157, and PA7, G265, G268, G269, G270, G271, G272, G273) with two substitutions in the fluoroquinolone targets (T83I in GyrA, and S83L/S83W/L95Q in ParC) reduced their wide range of ciprofloxacin resistance (MIC 8–128 mg/L) in presence of PAβN (MIC < 0.06-16 mg/L). These results indicate the presence of efflux-pump overexpression or the membrane permeabilization in those strains.

The mutational resistome of genes involved in the four fluoroquinolone efflux pump systems (MexA-MexB-OprM, MexC-MexD-OprJ, MexE-MexF-OprN and MexX-MexY-OprM) and their regulators and activators was analyzed in the 59 strains of our study (Table S2). The overexpression of efflux pumps plays a prominent role in the multidrug resistance of *P. aeruginosa,* and is mainly associated with the loss of function of their repressor genes (*mexR, nalC, nalD, nfxB, mexS, mvaT* and *mexZ*) or the gain of function of their activator genes (*mexT*) [3,14]. 

Non-synonymous mutations, deletions or insertions have been observed in all genes analyzed (Table S2). Alterations were most frequently observed in *mexD, mexX, mexY* and *nalC* genes (98, 98, 95 and 85% of the strains, respectively), and the lowest ones in *mvaT, mexT* and *mexA* genes (2, 8 and 10% of the strains, respectively). Multiple alterations in these chromosomal resistance genes were classified as wildtype *P. aeruginosa* natural polymorphisms [19], or were already reported showing no effect on efflux pump expression and considered as insignificant for the fluoroquinolone resistance, such as V126E in MexR, or G71E and S209R in NalC [20,21,22,23]. Thus, Figure 1 shows the alterations (premature stop codons, substitutions, frameshifts, deletions or insertions) previously reported as fluoroquinolone relevant ones [3,15,19,21,22,23,24] that were detected in the 59 *P. aeruginosa* strains of our study (Table S2). 

None of the previously reported mutations resulting in deficient NfxB or MexS activity as well as overexpression of MexEF-OprN or MexCD-OprJ efflux pumps, and associated with high-level (clinical) fluoroquinolone resistance [19,25,26,27], were detected among the 59 strains analyzed.

Regarding the nine strains with the two substitutions in GyrA and ParC targets, it was highlighted that the four strains (G268, G269, G270, PA7) with the highest ciprofloxacin MICs showed premature stop codons or deletions in MexZ (repressor of MexXY-OprM), and the remaining strains, with the exception of the G157 strain, (G265, G271, G272, G273) showed premature stop codons or deletions in the *nalD* gene (repressor of MexAB-OprM) (Figure 1, and Table S2). Those detected mutations lead to defective proteins which could not act as repressors in MexXY-OprM and MexAB-OprM pumps, respectively. Both efflux pumps are constitutively expressed in wild-type strains, but it has been reported that the loss of function of MexZ and NalD lead to efflux overexpression, respectively, and contribute modestly to clinical fluoroquinolone resistance [24,28].

On the other hand, it is interesting to highlight that the G157 strain, which was characterized by the reduction of >233 folds of ciprofloxacin MIC in PAβN presence (from resistant to susceptible levels), lacked the *mexX*, *mexY* and *mexZ* genes (Figure 1, and Table S2). PAβN is a broad spectrum efflux pump inhibitor, but its membrane permeabilization activity has also been described at concentration ≥16 mg/L in *P. aeruginosa,* being relevant in efflux pumps deficient strains [29,30,31]. The efflux pump deficiency linked to the PAβN ability to permeabilize membranes could explain the reduction to ciprofloxacin susceptibility in the G157 strain, and to our knowledge, this is the first detection in a clinical (no laboratory) *P. aeruginosa* strain.

### 2.3. TMQR Acquisition: Presence of CrpP

Forty-three strains (72.9%; 24 clinical, 17 non-clinical, and PA7 and UCBPP-PA14 reference strains) harbored the *crpP* gene, but no other TMQR was found in any of the 59 *P. aeruginosa* strains of our study (Figure 1, and Table S2). This *crpP*-carrying percentage was higher than those obtained in previous studies performed with clinical *P. aeruginosa* from Portugal (59.5%) [4], from India and Australia (63%) [5], from France and Switzerland (46%) [8] or from China (25.4–53.5%) [10,32], but lower than those previously described in clinical *P. aeruginosa* from Spain (84.6%) [4]. 

**Figure 2:**
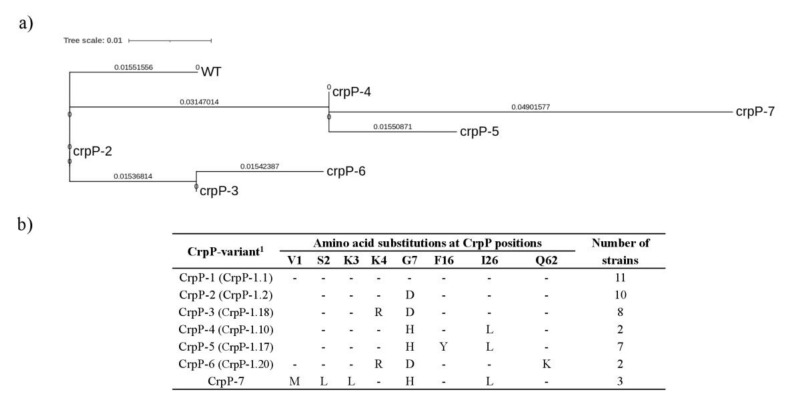
(**a**) Maximum-likelihood phylogenetic tree generated by the alignment of the amino acidic sequences of the CrpP variants found in this study. The alignment and the tree were generated with MEGA11. WT is CrpP-1. (**b**) Amino acid substitutions of each CrpP variant detected in this study compared to the first described CrpP in pUM505 plasmid (named CrpP-1) (GenBank accession number HM560971). ^1^ The variants included into brackets correspond to the variants previously detected by Zhu et al. (2021) [10]. CrpP-7 is a new variant found only in our study.

The amino acid sequences of CrpP of all 43 strains were compared with the first CrpP described (NCBI Reference Sequence: WP_033179079.1) [6], and seven different CrpP variants were found, grouped in three clades (Figure 2a). CrpP-1 corresponded with the wild-type protein, but the remaining six CrpP variants presented amino acid changes at least in positions Gly7 and/or Ile26 (Figure 2b). All the variants were previously found [4,9,10,32], except the variant CrpP-7, that has been described for the first time in this study. CrpP-7 showed 5 amino acid changes with respect to CrpP-1 (92% identity), and was detected in three fluoroquinolone-susceptible *P. aeruginosa* isolated from wild boar fecal samples. CrpP-1 was the predominant variant (11 strains, 26%), followed by CrpP-2 (10 strains, 23%), CrpP-3 (8 strains, 19%) and CrpP-5 (7 strains, 16%). The *crpP* gene was found in a wide variety of *P. aeruginosa* clones. Although no significant differences were observed between CrpP variant and sequence types, CrpP-4, CrpP-6 and CrpP-7 variants were detected in strains belonging to ST274, ST217 and ST1711, respectively, and the four strains belonging to high-risk clone ST175 harbored the CrpP-2 variant (Figure 1, and Table S2).

Since the CrpP-1 was initially identified in Mexico in 2018 [6], more than 37 CrpP variants have been reported in *Pseudomonas spp.* from at least 16 countries [4,9,10]. However, the *crpP* presence is not always associated with ciprofloxacin resistance. Indeed, twenty-one out of our 43 *crpP*-positive strains (53%) were susceptible to all fluoroquinolones tested, and the *crpP* presence did not significantly increase the fluoroquinolone MICs in our strains. Previous works also observed these results [4,5,8,10,27,32], highlighting a recent study where rigorous experimental results evidenced that CrpP is not a ciprofloxacin-modifying enzyme nor confers clinical fluoroquinolone resistance [33]. 

Independently of the impact on fluoroquinolone susceptibility, the global dissemination of *crpP* and homologous genes was observed, not only in *Pseudomonas spp*., but also in other Gram-negative bacteria genomes worldwide [7,9,11]. Indeed, *crpP* was originally identified in the conjugative plasmid pUM505 [6], although ICEs have been identified as the major reservoirs of *crpP* genes in *P. aeruginosa* [4,10,11]. The location of *crpP*-like genes into mobile genetic elements is of concern, because they also carry genes encoding virulence factors or involved in heavy metal resistance that can be spread among pathogens in different settings.

The analysis of the *crpP* genetic location in the genome of our *P. aeruginosa* strains revealed that the gene was located in chromosomal ICEs in all the 43-*crpP* positive strains. All *crpP* ICEs carried a MOBH2 relaxase, the ICE sizes ranged from 80 to 165 Kb with a G+C content of 59–60%, and tRNA-Lys was the ICE insertion site. The *crpP* gene was flanked by the highly conserved 45 bp direct repeats (*attL* and *attR*), and at both extremities by the *xerD* and *parA* genes, encoding an integrase of the tyrosine recombinase family and the plasmid partition protein A, respectively, as previously reported [8,10,11]. 

**Figure 3:**
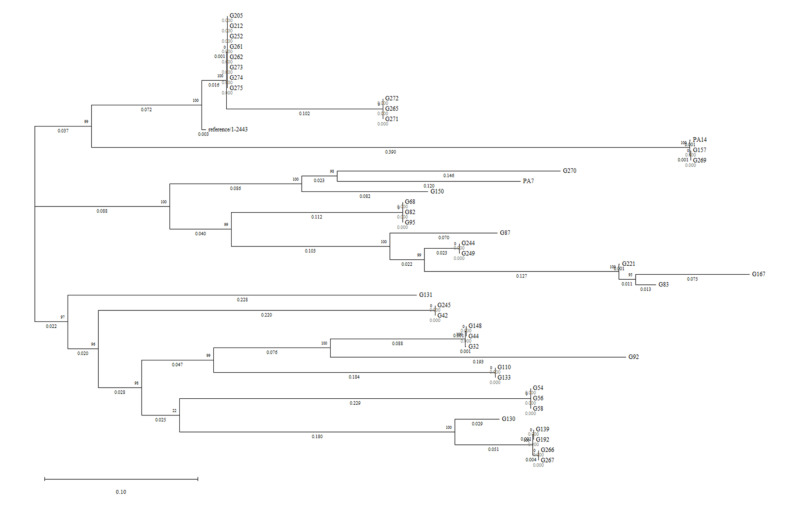
Phylogenetic tree of the *crpP*-ICEs detected in the 43 *crpP*-positive *P. aeruginosa* and the reference plasmid pUM505 (reference 1-2443) build based on the concatenated alignment of the high-quality SNPs, using CSI Phylogeny and MEGA-11.

The ICE structures of the 43 *crpP*-positive *P. aeruginosa* strains, as well as the reference plasmid pUM5053 were analyzed. The *crpP*-ICEs were variable in composition, although displaying a similar backbone gene organization. A phylogenetic tree was constructed from the core single-nucleotide polymorphisms (SNPs) within the ICE backbones (using the sequence of pUM505 as reference) (Figure 3). Three separate clustering groups and 23 different ICEs were observed. 

**Figure 4:**
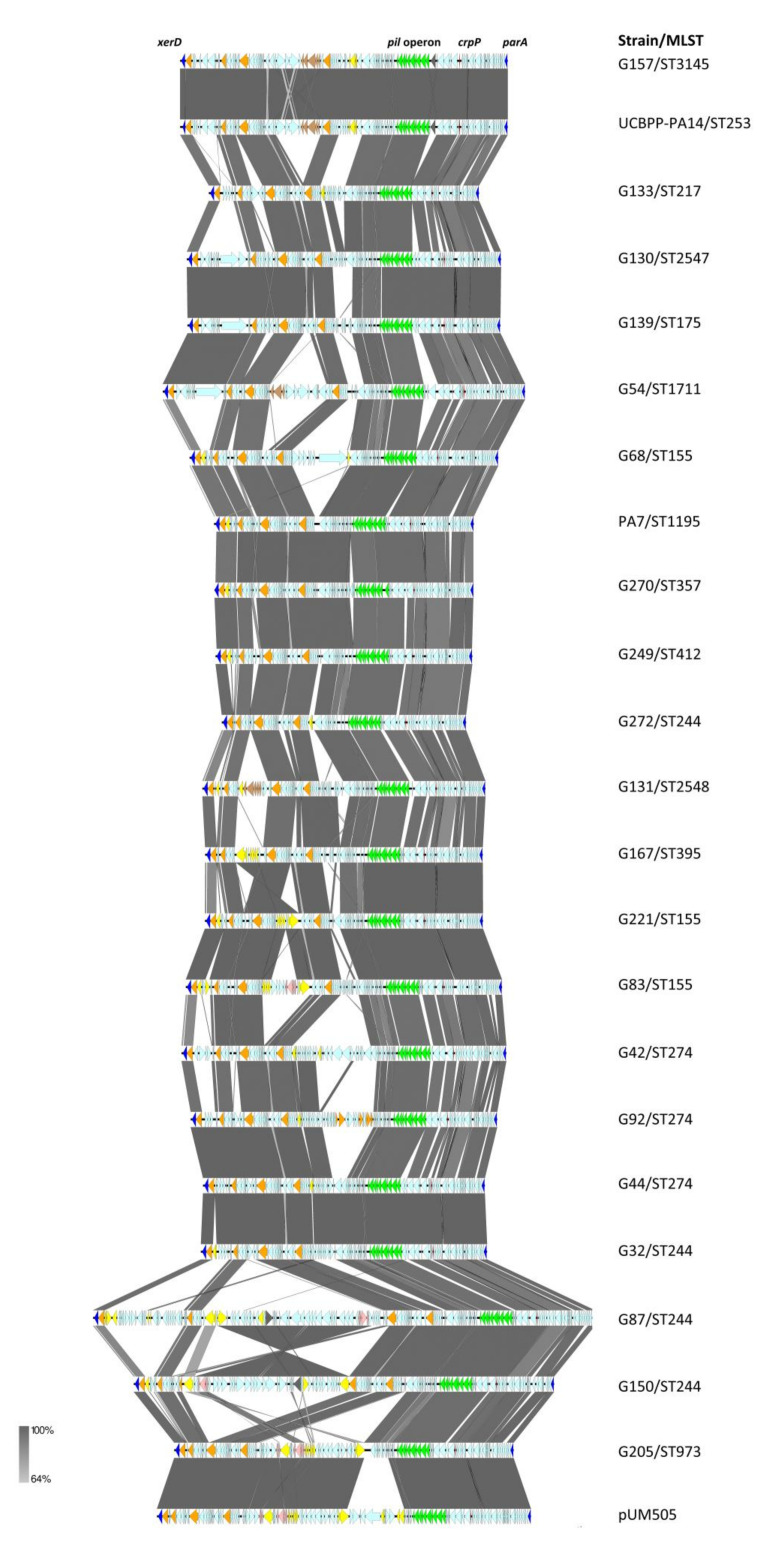
Comparison of different genetic environments of *crpP*-harbouring *P. aeruginosa*. Arrows represent genes and indicate the orientation. Relevant genes are indicated by colored arrows as follows: *xerD* and *parA* genes (dark blue), mobilization genes (*virB, virD*, *tra* genes and *trb* operon) (orange), *pil* operon (green), *mer* operon (pink), fimbrial operon (brown), mobile element (IS and Tn) (yellow), pyocin gene (dark grey) and *crpP* gene (red).

Some association has been observed between the type of *crpP*-ICE and the *P. aeruginosa* sequence type (ST), highlighting that all *P. aeruginosa* ST973 (G205, G212, G252, G261, G262, G273, G274 and G275) showed the same *crpP*-ICE (Figure 3). The G157, G269 and UCBPP-PA14 strains belonged to the clonal complex CC253 and harbored similar *crpP*-ICEs. However, the strains belonging to ST155, ST244 and ST274 had a great variety of *crpP*-ICEs. The genetic structures of the 23 different *crpP*-ICEs identified in Figure 3 were compared, and all of them showed a conserved and a variable region (Figure 4). The conserved region (delimitated between the class I SAM-dependent methyltransferase encoding gene and *parA*) included the *crpP* gene and the type IV pili synthesis operon (*pil* operon). On the other hand, a great variety of structures was detected in the variable region (delimitated between *xerD* and the class I SAM-dependent methyltransferase encoding gene) of the 23 different *crpP*-ICEs. Several insertion sequences (*ISPa32*, *ISPa40, ISPa97*, *ISPa121, ISPa125, TnAs1*, etc.), the fimbriae related *cupA*, *cupD* or *cupE* clusters, the mercury resistance *mer* operon, the pyocin S5 and S8 bacteriocin encoding genes, and mobilization genes such as *virB*, *virD*, *tra* and *trb* operon, among others, were included in the *crpP*-ICE variable regions (Figure 4). Among these elements found in the *crpP*-ICEs, *cup* locus encodes bacterial adhesive organelles, and *pyoS5* and *pys8* genes encode S5 pyocin and S8 pyocin, respectively, that mediate bactericidal activity against other *P. aeruginosa* isolates were detected. The genetic environment of *pys8* is organized in transposon Tn*6350* [34], being this structure found in the strains G87 and G150 within *crpP*-ICE. Additionally, the 23 integrases XerD identified in this study shared from 59.76 to 99.77% amino acid identity, and their protein alignment was plotted in a tree where four different branches were identified (Figure S1).

The presence of this arsenal of genes, together with the fact that ICEs have the capacity to be transferred among *P. aeruginosa* strains, highlights the *crpP*-ICE role in the dissemination of resistance mechanisms and virulence that would enhance, on host *P. aeruginosa* strains, the adaptation to different niches, including hospital environment, as well as its expansion towards other relevant genera (i.e., *Acinetobacter*).

**Figure 5:**
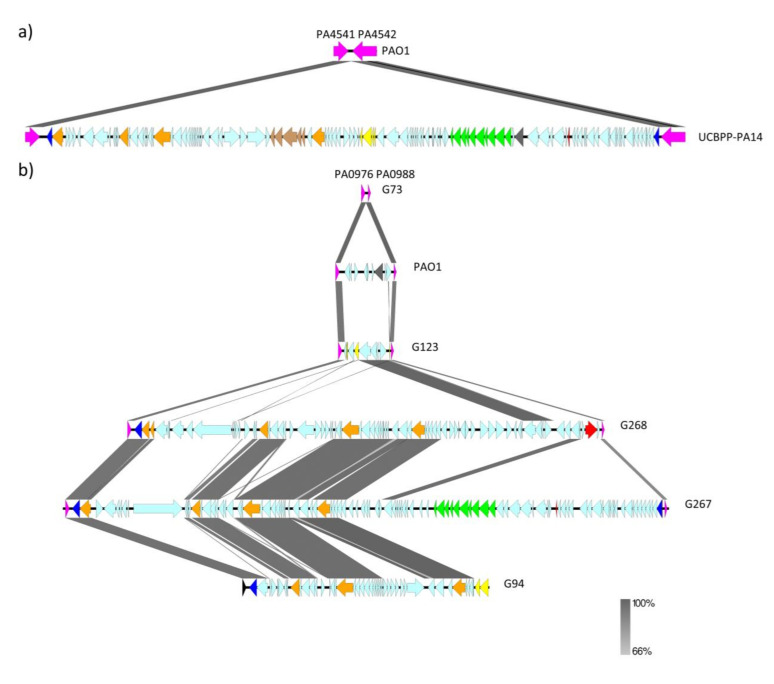
Mobile elements inserted after the tRNA-Lys of *P. aeruginosa* genome: a) PA4541/PA4542 (*lepA/clpB*) empty region in PAO1 strain and *crpP*-ICE inserted in UCBPP-PA14 strain. b) PA0976/PA0988 (*queC*/CoAhydrolase) empty region in G73 strain, *pyoS5*-ICE in PAO1 strain, PAGI-5-like in G123, *exoU*-PAGI in G268, *crpP*-ICE in G267, and an ICE removing from PA0988 to PA0998 in G94. Relevant genes are indicated by colored arrows as follows: *xerD* and *parA* genes (dark blue), mobilization genes (*virB, virD, tra* genes and *trb* operon) (orange), *pil* operon (green), fimbrial operon (brown), mobile element (*IS* and Tn) (yellow), pyocin gene (dark grey) and *crpP* and *exoU* genes (red).

The *crpP*-ICEs were integrated into specific hotspots. The tRNAs are known to serve as integration sites for ICEs and phage-like elements [35]. Indeed, all the 43 *crpP*-ICEs detected in this study were integrated at the end of the tRNA-Lys that was found alone or in a tRNA cluster (tRNA-Asn, tRNA-Pro, tRNA-Lys), as was previously described [11]. The two different genomic environments detected (PA4541/PA4542 and PA0976/ PA0988) were as follows: i) PA4541/PA4542, the ICE was inserted between PA4541 (*lepA*) and PA4542 (*clpB*) after the tRNA cluster (observed in 29 strains, including UCBPP-PA14); or ii) PA0976/PA0988, ICE inserted between PA0976 (*queC*) and PA0988 (CoAhydrolase) after the single tRNA-Lys (detected in 14 strains, including PA7) (Figure 5, Table S4). These two insertion points are considered as regions of genome plasticity (RGPs) [35]. Interestingly, all the 14 strains harboring the *crpP*-ICEs inside the PA0976/PA0988 point, showed the “hot spot” PA4541/PA4542 empty. However, different structures were observed in the 29 strains with the *crpP*-ICEs inserted in the PA4541/PA4542 point: 10 strains showed an empty PA0976/PA0988 structure, 4 strains carried the *exoU*-PAGI, 6 strains harbored an ICE with the *pyoS5* gene, one strain presented a genomic island similar to PAGI-5 (GenBank accession No. EF611301), and the PA0976/PA0988 region was not totally ascertained in 8 strains (Figure 5, Table S4). 

The presence of the PA4541/PA4542 and PA0976/ PA0988 insertion points was also investigated in the 16 *crpP*-negative *P. aeruginosa* strains of this study (Figure 5, Table S4). The PA4542/PA4541 insertion point was empty in all but one strain. The PA0976/PA0988 region was empty in six strains, but included the *exoU*-PAGI in two strains, the *pyoS5*-ICE in four strains (including PAO1 strain), the PAGI-5-like in two strains, and an ICE in two strains, whose insertion removed from PA0988 to PA0998 region, leaving a partial *pqsB* gene (PA0997) (Figure 5, Table S4). All these elements, including the *crpP*-ICE, belong to the pKLC102-subtype genomic islands, which harbor a XerC/XerD integrase gene. These integrases recognize these two insertion points which both are the unique tRNA-Lys present in the *P. aeruginosa* genome [35].

In summary, resistance to fluoroquinolones in *P. aeruginosa* is multifactorial as Table 1 shows. Genomic information could predict ciprofloxacin resistance and susceptibility, such as recent works have reported using manual and machine learning approaches [19,36]. Particularly, mutations in the QRDR of GyrA and ParC have the highest effect on fluoroquinolone resistance levels. In our study, other related proteins were found to be altered or absent in resistant strains, such as those implicated in efflux pumps regulation: MexZ, MvaT and NalD. Other alterations in the studied genes were detected, but in susceptible- and resistant-strains, and therefore, their implication in resistance could not be proved. The same fact occurs with the presence of CrpP variants. We have reported the spread of *crpP*-like genes in a large series of *P. aeruginosa* obtained from a clinical and non-clinical origin. Despite CrpP not conferring fluoroquinolones resistance per se, the emergence of other CrpP variants as well as in combination with other mechanisms may be considered and monitored. Furthermore, the location of *crpP*-like genes in mobilizable ICEs in *P. aeruginosa* is of significant concern, because genes encoding resistance to heavy metals and virulence factors are coharbored. 

This work provides a genetic explanation of the fluoroquinolone resistance and *crpP*-associated pathogenesis of *P. aeruginosa* recovered from clinical and healthy humans, healthy animals, environmental, and food samples. A reduction of fluoroquinolone use, a continued monitoring of evolving antibiotic resistance patterns, and novel research approaches including new antibiotics with novel modes of action, phage therapy or antisense agents, are required to minimizing the impact of fluoroquinolone resistance in *P. aeruginosa*.

## 3. Materials and Methods

### 3.1. Bacterial Strains and Fluoroquinolone Susceptibility Testing

A total of 56 *P. aeruginosa* strains were selected from the *Pseudomonas* collection of the Molecular Microbiology Area (Centre for Biomedical Research in La Rioja, CIBIR, Logroño, Spain) (Table S1). These strains were obtained from clinical (*n* = 28) and non-clinical (*n* = 28) samples from Spain. *P. aeruginosa* PAO1, PA7 and PA14 reference strains were also included in the study.

Minimum inhibitory concentrations (MIC) of ciprofloxacin, levofloxacin, norfloxacin and ofloxacin (Sigma-Aldrich, St. Louis, MO, USA) were determined by agar dilution method using *P. aeruginosa* ATCC27853 strain as control strain and interpreted according to CLSI (2020) [13]. Furthermore, to assess the role of efflux pumps, MIC of ciprofloxacin was determined in presence and absence of the inhibitor Phe-Arg-β-naphthylamide (PAβN, 40 mg/L, Sigma-Aldrich, St. Louis, MO, USA). Isolates were defined as efflux pump overproducers when MIC_without PAβN_/MIC_PAβN_ was > 2, as previously described [18].

### 3.2. Whole Genome Sequencing (WGS)

Genomic DNA was extracted using the Wizard^®^ Genomic DNA Purification Kit (Promega, Madison, WI, USA). Quantity and quality were evaluated using a Qubit fluorimeter (Thermo Fisher Scientific, Waltham, MA, USA). Libraries were prepared using the TruSeq DNA PCR Free protocol (Ilumina, San Diego, CA, USA). Then, the final libraries quality was assessed with Fragment Analyzer (Std. Sens. NGS Fragment Analysis kit 1- 6000 bp), and quantified by qPCR at the Genomics and Bioinformatics Core Facility (CIBIR). Subsequent sequencing was carried out in an Illumina HiSeq 1500 (Illumina, San Diego, CA, USA). 

FastQC (https://www.bioinformatics.babraham.ac.uk/projects/fastqc/) was used to analyze the quality of raw reads, which were subsequently trimmed and filtered by using Trim Galore (https://www.bioinformatics.babraham.ac.uk/projects/trim_galore/). Genomes were reconstructed using PLACNETw [37] or PATRIC [38]. Identification of Open Reading Frames (ORFs) and genome annotation of the assembled genetic elements was performed by PROKKA v1.13 [39].

The genomes of *P. aeruginosa* PAO1, UCBPP-PA14 and PA7 strains, and the pUM505 plasmid sequence were downloaded from the NCBI database (GenBank accession No. GCF_000006765.1, GCF_000014625.1, GCF_000017205.1 and HM560971, respectively).

### 3.3. Resistome

The presence of acquired fluoroquinolone resistance genes was evaluated using ResFinder v4.1 [40,41,42]. The *crpP* gene was confirmed using Blastn tool, and the mutations were determined by comparison with *Pseudomonas aeruginosa* pUM505 *crpP* gene (GenBank accession No. NG_062203.1) using the Clustal Omega tool [43]. 

The mutational resistome was determined by analyzing the mutations in 23 chromosomal genes implicated in fluoroquinolone targets and efflux pumps (Table S3) using Clustal Omega tool, and compared with *P. aeruginosa* PAO1 genes from Pseudomonas Genome Database (https://www.pseudomonas.com) [44]. 

### 3.4. Genetic Context of crpP

The genetic environment of the *crpP* gene was investigated. The *crpP*-containing ICEs were reconstructed and reannotated manually using Artemis and Mauve [45,46]. A comparative map was drawn using Blast (V2.10) and Easyfig tools [47]. The pUM505 plasmid sequence was considered as reference. 

The genetic relationship in this region was analyzed in all strains by CSI Phylogeny, Mega 11 and iTOL [48,49,50].

## Figures and Tables

**Table 1:** Fluoroquinolone Resistance Levels and Associated Changes Detected in the Strains included in this Study.

Number of Strains	Fluoroquinolone MIC (mg/L) ^a^				Fluoroquinolone Relevant Modifications Detected in [nºstrains] ^b^	CrpP Variant (nºstrains) ^c^
	CIP	LEV	NOR	OF		
8	64–256 (R)	128–256 (R)	128–512 (R)	128–512 (R)	GyrA (T83I; D87N); ParC (S87W) (4) GyrA (T83I); ParC (S87W); MexZ (ΔV102-V105) (1) GyrA (T83I); ParC (S87L); MexZ (V43*/ E98fs) (2) GyrA (T83I); GyrB (I529V); ParC (S87L); MvaT (ins_ntG66); MexZ (Δnt216-225) (1)	CrpP-2 (4) CrpP-3 (1) CrpP-2 (1); None (1) CrpP-2 (1)
5	8–16 (R)	4–128 (R)	64–128 (R)	16–128 (R)	GyrA (T83I); ParC(S87L); NalD (R164*) (3)	CrpP-1 (3)
					GyrA (T83I); ParC (L95Q); ParE (A473V); NalD (V151fs) (1)	CrpP-1 (1)
					GyrA (T83I); ParC (S87W); ΔMexXYZ (1)	CrpP-3 (1)
7	2–4 (R)	4–16 (R)	8 (I)	8–16 (R)	GyrA (T83I); ParE (A473V/ H461D, A473V) (3)	CrpP-1 (3)
					GyrA (T83I); MexB (Δnt2131-2143) (1)	CrpP-5 (1)
					ParE (A473V) (2)	CrpP-1 (2)
					MexX (Q85*) (1)	CrpP-5 (1)
1	1 (I)	16 (R)	8 (I)	16 (R)	GyrB (S466F) (1)	None (1)
1	1 (I)	1 (S)	1 (S)	1 (S)	MexT (P28fs) (1)	None (1)
2	0.5 (S)	2 (I)	4 (S)	4–8 (I-R)	GyrB (S466A) (2)	CrpP-5 (2)
35	0.06–0.5 (S)	0.125–2 (S-I)	0.125–8 (S-I)	0.25–4 (S-I)	ParE (A473V) (2)	CrpP-6 (2)
					ParE (A473V); MexZ (ΔE21-G28) (2)	CrpP-1 (2)
					GyrB (I529V) (6)	CrpP-7 (3); CrpP-3 (1); None (2)
					MexF (Q178*) (2)	CrpP-2 (2)
					OprM (P173fs) (1)	CrpP-4 (1)
					WT (22)	CrpP-2 (2); CrpP-3 (5); CrpP-4 (1); CrpP-5 (3); None (11)

^a^ Breakpoints according to CLSI 2020 [13]. CIP: ciprofloxacin; LEV: levofloxacin; NOR: norfloxacin; OF, ofloxacin. ^b^ Amino acid changes compared to PAO1 as reference strain, except for MexT which was compared to UCBPP-PA14 reference strain. The slanted line (/) separates different patterns of modifications. Abbreviations: *, premature stop codon; fs, frameshift; ins_nt, nucleotide insertion; Δ, deletion; ^c^ CrpP-1 allele corresponds to the wild-type protein described in pUM505, and the remaining CrpP variants showed the following changes: CrpP-2 (G7D); CrpP-3 (K4R, G7D); CrpP-4 (G7H, I26L); CrpP-5 (G7H, F16Y, I26L); CrpP-6 (K4R, G7D, Q62K); CrpP-7 (V1M, S2L, K3L, G7H, I26L).

## Data Availability

All datasets are available. The whole genome data for all 56 *P. aeruginosa* strains have been deposited at GenBank using the BioProject numbers PRJNA526213, PRJNA526344, PRJNA528628, and PRJNA796464. Supplementary Table S5 shows the accession numbers for sequences of *P. aeruginosa* strains.

## Supplementary Materials

The following supporting information can be downloaded at: https://www.mdpi.com/article/10.3390/antibiotics11091271/s1, Table S1: Characteristics of the 56 *Pseudomonas aeruginosa* strains isolated from different origins; Table S2: Minimum inhibitory concentrations (MIC) of fluoroquinolones, CrpP variants and fluoroquinolone mutational resistome detected in the 59 analyzed *Pseudomonas aeruginosa* strains; Table S3: Dataset and function of the 23 antimicrobial resistance genes analyzed to determine the mutational resistome of the *P. aeruginosa* strains; Table S4: Mobile elements inserted in PA0976/PA0988 and PA4541/PA4542 insertion points of the 59 analyzed *Pseudomonas aeruginosa*; Table S5: Accession numbers for *Pseudomonas aeruginosa* strains in this study; Figure S1: Maximum-Likelihood phylogenetic tree generated by the alignment of the amino acidic sequences of the XerD integrases found in this study.
